# Patent foramen ovale closure

**DOI:** 10.4103/0974-2069.64372

**Published:** 2010

**Authors:** Zahid Amin

**Affiliations:** Professor and Director, Cardiac Catheterization and Hybrid Suites, RUSH Center for Congenital and Structural Heart Disease, RUSH Univeristy Medical Center, Chicago, Illinois, USA

## INTRODUCTION

The technical considerations for closure of patent foramen ovale (PFO) are simple, it is one of the more common procedures that is performed by the interventional cardiologists, the learning process is straightforward, the devices available for closure are several, the risks to the patient are relatively low, and off and on it has been found culprit in devastating complications attributed to its presence. With simple introduction as above, it appears as an open/shut case! However, majority of the statements made above are anecdotal, there are no scientific data that may prove that PFO is absolutely the culprit in most of the instances, if not all.[1-3]

PFO is one of the most common congenital defects and hence,[4] the significant interest of the industry in developing devices for closure is understandable. Clinically, a huge advantage of industry interest is that expensive trials (sponsored by the industry) can be conducted which may incriminate PFO as a factor in cerebrovascular accident, migraine and transient ischemic attacks.[1,5-11]

Keeping in mind the title of the article, I would like to focus on the technical aspects of the procedure based upon the interventional anatomy of the PFO and the controversy that surrounds its closure.

## ANATOMY OF THE PFO FROM THE INTERVENTIONALIST'S PERSPECTIVE

PFO may as well stand for “potential for opening” in addition to patent foramen ovale. In majority of the population, it is a communication that has closed completely or remained closed with potential to open under altered physiological conditions. The communication is a flap type opening where the septum primum (the thin septum, the frail septum, the fluttering septum, the aneurysmal septum) keeps it closed until and unless the pressure on the right atrial side increases transiently (as in straining, coughing, sneezing). If the septum primum coaptation to the septum secundum (the thick septum, the limbus, the superior vena cava (SVC) rim, the aortic rim) is lost because of atrial enlargement, the communication becomes a true opening.

**Figure 1:**
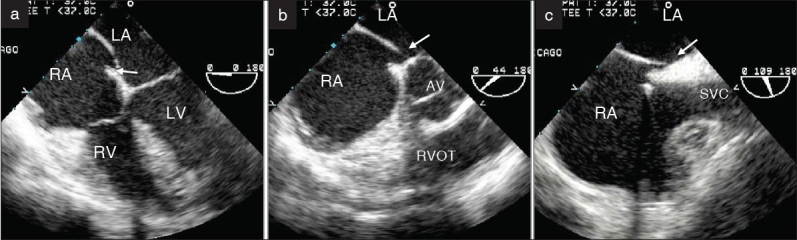
Transesophageal evaluation of PFO. (a) Four-chamber view to evaluate the atrioventricular valves and superior rims. (b) Aortic short-axis view to evaluate the aortic and the posterior rim and (c) Bi-caval view to evaluate the superior vena cava and the inferior vena cava rims. Note that the septum primum (the thin septum) bulges into the left atrium in the four-chamber view

**Figure 2:**
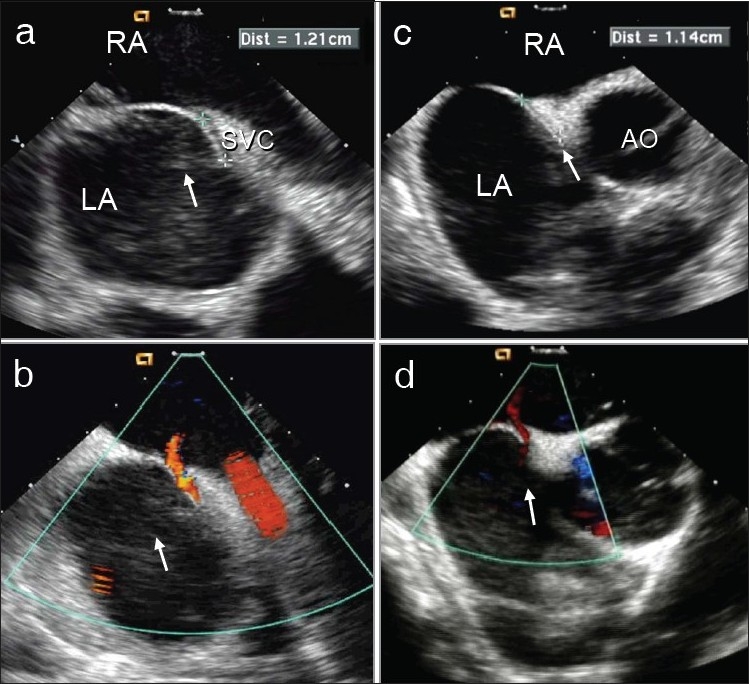
Intracardiac echocardiography documenting two standard views. (a,b) is the bi-caval view without and with color, respectively. This is a tunnel PFO with significant overlap of the septum primum on the septum secundum (the SVC rim). The measurements in the picture is of the length of the tunnel. (c,d) Aortic short-axis view without and with color, respectively. Aortic rim is seen with septum primum overlapping the septum secundum (the aortic rim).

Although the communication is a flap type opening, its shape is dependent upon the length of coaptation between the edge of the septum primum and secundum [Figures 1 and 2]. Generally, the opening is small and behind the ascending aorta. The opening may increase in dimension (antero-posterior dimension) and become lacunar type if the septum primum remains separated from the secundum. The opening may extend posteriorly toward the SVC. Hence, PFOs can come in many different sizes although they appear small if examined in one dimension only. These findings prompted physicians (including myself) to always balloon size PFO before placing a device. The degree of overlap between the septum primum and secundum determines whether the PFO is simple or tunnel type. If the primum septum overlaps significantly, the PFO opening is called tunnel-type opening and the direction of the tunnel is infero-superiorly. The presence of tunnel in a PFO adds another twist and increases the complexity of the PFO and has generated more debate than expected (vide infra).

Another important consideration while evaluating PFOs is the consistency of the primum septum. If it is thin, frail and its excursion is 15 mm or more (in some literature 10 mm or more), it is termed as aneurysmal PFO. The aneurysmal nature of the tunnel adds to PFO complexity as closure of tunnel type of PFO is difficult and no particular device appears to be just right.

## THE CONTROVERSY BEHIND CLOSURE OF PFO

### Paradoxical embolism

There have been numerous reports that have been published over the last decade that have either tried to prove PFO as the cause of cerebrovascular accident (CVA) with probably equal number of reports that contradict the notion.[2,3] At the current time it is difficult to prove or to rule out the involvement of PFO in cryptogenic stroke. There are, however, several industry-sponsored, Food and drug Administration (FDA)-approved studies underway that are prospective, randomized, carefully planned and will be statistically valid.[1] The answer, hence, is not too far in the future.

Regardless, if I am to close a PFO, my primary objective should be to optimally close the defect with all the above-mentioned anatomical factors of PFO in mind, to choose a device that will fit the defect in the best possible manner and will achieve complete closure. The PFO is closed in patients with increased risk of recurrent paradoxical embolism, not for volume overload as is the case in patients with moderate to large atrial septal defect (ASD). While a small residual shunt in patients with ASD is of no hemodynamic significance, a small residual shunt in patients with PFO may carry much higher stakes; these patients undergo the procedure to get rid of a small shunt at the atrial level hence if a small shunt remains, the procedure should be considered a clinical failure.

## HOW I CLOSE IT?

### Routine right heart catheterization

Although these patients do not have volume overload, a right heart catheterization is highly recommended in these patients. Evaluation of pulmonary artery pressures may preclude closure in a very small subset of patients with severe pulmonary artery hypertension where PFO may be acting as a pop-off mechanism and maintain cardiac output at the expense of low systemic saturations. In our institution, we perform right heart catheterization in all patients prior to PFO closure. Heparin (100 units/kg) is given intravenously once the pulmonary arterial saturations have been obtained. At the conclusion of the procedure, it is highly recommended that protamine is not given to reverse the effect of heparin.

### Defect assessment

Either trans-esophageal echocardiography (TEE) or intra-cardiac echocardiography (ICE) is performed during the procedure. In my opinion, PFO is a echocardiography-driven procedure. A complete assessment of the type of the defect, shape of the defect, placement of device and post-procedure evaluation is crucial, and the importance of using echocardiography cannot be emphasized enough.

By using TEE or ICE, all septal rims need to be evaluated thoroughly.[12,13] If the procedure is to be performed by an Amplatzer (AGA Medical, Plymouth, MN) device, it is recommended to measure the aortic and the SVC rims. These measurements are important for size selection of the device. According to the instructions for use pamphlet, if the aortic or the SVC rim is smaller than 9 mm, Amplatzer PFO occluder should not be used. The smallest PFO device available is 18 mm in diameter and 9 mm in radius, and hence the recommendations of not placing the device if the aortic or the SVC rims are smaller than 9 mm. Generally, the SVC is almost always adequate in size but the aortic rims are smaller than 9 mm in a substantial number of cases. The device, however, has been used in patients with smaller aortic rims without any sequelae.[9] These measurements are erroneous as the aortic rim deficiency or sufficiency is always relative. For example, while performing TEE, aortic rim may be completely absent in 30° angulation but present in 45° to 65. Aorta surrounds the atrial septum from the antero-inferior to superior port of the atrial septum and it is not possible to have completely deficient rim throughout the course of the aorta in patients with PFO. The idea behind 9 mm aortic or SVC rims was to protect the aorta and the left atrial free wall from injury if the edge of the device was in contact with either. As per experience, there are other factors rather than mere contact of the device edge that usually lead to complications.[9,10] Unfortunately, these measurements do not take into account the complex anatomy of the PFO (vide supra).

Occasionally, pre-procedure studies merely suggest the presence of PFO and hence it is important to document shunt (right to left) in the cardiac catheterization laboratory. Physicians usually perform saline contrast echocardiography to document right to left shunt. The catheter is placed in the right atrium and agitated saline is injected rapidly through the catheter. At the same time, valsalva maneuver is also performed to facilitate right to left shunting. Generally, we press on the patient's abdomen while asking him/her to breathe in. There are other maneuvers that can be performed if the PFO needs to be documented or when PFO cannot be crossed after several attempts. One performs the bubble study with the catheter in the SVC; this helps in localizing defects that are superiorly located and toward the SVC. In a few situations, I have advanced the catheter so as to tent the primum septum, once the septum is tented, saline contrast injection is performed. This maneuver tends to separate the septum primum from the septum secundum and bubbles cross into the left atrium rather easily. It is imperative that bubbles be documented in the left atrium within the first three beats of injection as after more than three beats, it is difficult to ascertain if the bubbles are coming from the pulmonary veins.

### Balloon sizing

For Amplatzer devices, balloon sizing is not recommended. I, however, recommend balloon sizing in all patients for several reasons. Firstly, it helps in defining the size of PFO which may not be evident by routine measurements. Secondly, it helps to define the length of the tunnel. Thirdly, it helps to rule out other defects. Fourthly, in patients with thin and aneurysmal septum, it defines the septal anatomy by decreasing the extent of motion of the atrial septum. For the Helex (W.L Gore and Associates, Flagstaff, AZ) device, balloon sizing is routinely performed and helps in proper size selection of the device. Use of the Helex device is an off-label use of the device since the device is approved for ASD closure. The diameter of the discs to defect ratio should be 1.7 or higher.

In the USA, there are no devices that are approved by the FDA for PFO closure. Several devices are available though the Amplatzer PFO device use is restricted to patients enrolled in the RESPECT trial (Randomized Evaluation of recurrent Stroke comparing PFO closure to Established Current standard of Care Treatment). The statistical design is “event driven", meaning that the total number of patients is not considered but only numbers of end point events are considered. There are four pre defined stopping rules for this trial, success will be declared if a positive stopping rule is reached (device is significantly better than current standard of care treatment).

The Helex device is being used under GORE REDUCE trial. The estimated completion date is 2014. The study objective is to demonstrate that PFO closure with the GORE HELEX Septal Occluder plus antiplatelet medical management is safe and effective and reduces the risk of recurrent stroke or imaging-confirmed transient ischemic attack (TIA) when compared to antiplatelet medical management alone in patients with a PFO and history of cryptogenic stroke or imaging-confirmed TIA.

Enrollment for CLOSURE I trial was completed recently. This trial was managed by the NMT technologies. The result of this trial will be available at the end of this year or in early 2011.

CLOSE (France) trial is designed to compare PFO closure with anticoagulation versus antiplatelet therapy to prevent stroke recurrence. Any device can be used in the trial. The estimated completion date is 2012.

PC-trial (Europe and Australia) is being done with Amplatzer PFO occluder. Anticipated completion date is 2011.

## THE PROCEDURE

I use a wedge catheter (end-hole catheter) of 7F diameter. The inner lumen of the catheter admits a stiff wire. In order to provide stiffness to the catheter, I routinely advance an Amplatzer super-stiff wire through it (advanced to the tip of the catheter-not outside of the catheter). I cross the PFO while coming from the inferior vena cava, a gentle clock-wise torque while advancing the catheter is helpful in crossing almost all defects. The primary trick is that the interventionalist should be able to propagate the torque while advancing the catheter in. Static rotation of the catheter without pushing or pulling it out of the body is not helpful, as any torque applied to the catheter has to be transmitted to the tip of the catheter, hence the term “propagate the torque."

Rarely, I have gone to the SVC and pulled the catheter tip in the right atrium to cross the PFO.

Sometime, a multi-purpose or Judkin's right coronary artery (St. Jude Medical, Mineapolis, MN) catheter can be used. A Terumo glide wire is very helpful in teasing the catheter through the PFO.

Once the catheter is across the PFO, a wire is advanced through it into the left upper pulmonary vein. This maneuver should be performed with absolute care. Anatomically, the location of the left atrial appendage and the left upper pulmonary vein are very close to each other. Under fluoroscopy, a wire in the left atrial appendage appears to be in the left upper pulmonary vein. If the wire is pushed further, it may perforate the atrial appendage[14,15] which may result in pericardial effusion and/or tamponade at the conclusion of the case (after the wire/catheter are removed). A catheter or wire in the appendage seems to move excessively as opposed to when it is in the pulmonary vein (fixed structure in the lungs); a catheter in the appendage causes premature atrial contractions as opposed to when it is in the pulmonary vein. Finally, if there is any question about the location of the catheter, a small hand injection of contrast can easily delineate the left atrial appendage anatomy.

Once, the wire is in the left upper pulmonary vein, the catheter is removed and the delivery sheath advanced over the wire. The sheath may or may not be advanced into the pulmonary vein. Its position can be easily checked with echocardiography. The dilator and the wire are removed (for the AGA device). The sheath is allowed to bleed back and then flushed with saline. The syringe is kept attached to the sheath and the device is prepared.

The device is advanced through the delivery sheath and under fluoroscopy and echocardiography, the device is deployed in the usual fashion.

Since the PFO is a slit-like opening, it is important that the right atrial disc overlaps the limbus. If there is no right atrial disc overlap, the risk of device embolization to the left atrial side increases. A few physicians have used fluoroscopy alone to close the PFO.[16] There are fluoroscopic signs that usually suggest that the device is in optimal location. In all of those cases, a thorough echocardiogram had been performed to rule out additional defects and estimate the size of the PFO. I believe that this practice be avoided at all costs. If we are going to make PFO closure a routine and bedside type of procedure, then perhaps we should use echo alone to close such defects. I believe that both modalities should be used for best possible outcome and patient safety.

## POST-PROCEDURE MANAGEMENT

Patient is started on Plavix (clopidogrel 75 mg) and either full dose or baby aspirin. The plavix can be discontinued after 3 months. The ASA is maintained for at least 6 months. Patients, who were on coumadin before the closure, are maintained on coumadin and aspirin after the procedure.

## TROUBLE SHOOTING

### Cannot cross the PFO

This is not an uncommon situation especially for the novice. First and foremost, ensure that the patient has a PFO. Saline contrast echocardiography should be performed with a catheter in the IVC and SVC. Valsalva maneuver should be performed during saline contrast echocardiography. Bubbles should appear in the left atrium in the first three heart beats after the injection. Torque propagation, while maneuvering the catheter is important. If the catheter is properly angulated, use of an angled glide wire can be very helpful in difficult cases.

The longer the length of the tunnel, the more difficult it will be to cross the defect. Multiple catheters with different angles and curves can be used.

In my experience of several hundred cases, I have used trans-septal puncture technique twice to cross the PFO. Some physicians believe that it is not a good practice to make a hole to close a hole. I agree with the philosophy. The puncture has to be in a precise location that will assure closure of PFO with the device.

### Cannot assess the length of the tunnel

Length of the tunnel can be assessed during balloon sizing. The length of the waist of the balloon, while inflating the balloon is very helpful. Some physicians have used a balloon tipped angiographic catheter. The catheter is advanced through the defect in the left atrium. The balloon is inflated and the catheter pulled back till it completely occludes the PFO. A small amount of contrast in injected. The side-holes of the catheter are situated in the tunnel and hence it gets opacified.

If the septum primum is thin and aneurysmal, a balloon-tipped catheter is inflated in the left atrium and pulled into the right atrium. This maneuver displaces the primum septum from the left atrial side to the right atrial side. The tunnel gets foreshortened and device discs oppose the septum better.

### Atrial ectopy with the catheter in the right/left atrium

Atrial ectopy with the catheter in the left atrium suggests that the catheter is either coming in contact with the left ventricle or is in the left atrial appendage. Ectopy with the catheter in the right atrium is usually related to the catheter position in the right atrial appendage. Needless to say, that the position of the catheter needs to be adjusted. Both atrial appendages are fragile and a gentle push of the catheter can perforate the appendage with resultant pericardial effusion.

### Device embolized to left atrium immediately after release

This happens for two reasons. First, the right disc did not cover the limbus and slid out of the defect after release and second, it never straddled the septum. Usually, the device migrates from the left atrium to the ventricle and thence to the aorta. The most difficult location for device retrieval is the left ventricle. If it gets stuck in the mitral valve apparatus, patient should be referred to surgery. If it is in the left ventricular cavity, left atrium or the aorta, it can be retrieved using percutaneously.

### Do's and Dont's of PFO closure

Do:

1. right heart catheterization
2. heparanize
3. assess all atrial septal rims
4. balloon size
5. ensure the right atrial disc straddles the limbus
6. keep patient in-house overnight
7. repeat echocardiogram the next morning
8. pay attention to chest pain/discomfort

Don't's

1. reverse heparin
2. flush the delivery sheath before it back bleeds under gravity
3. perform trans-septal puncture for cosmetic purposes
4. do routine dental procedure for 6 months
5. close PFO in patients with severe pulmonary hypertension
6. close PFO if not clinically indicated
7. panic if the device embolizes
8. try to retrieve the device if it is stuck in the left or right ventricle atrio-ventricular valve.

## SUMMARY

PFO closure is one of easiest procedure for the interventionalist. Its assessment before device closure is crucial. There are several devices available for PFO closure and hence several techniques can be used for closure of PFO. A device-based specific technique is preferable fro PFO closure. Regardless of the device type, a preliminary assessment of the atrial septum, type of PFO, and use of TEE or ICE are very important to have optimal outcome.
